# Orphan CpG Islands Identify Numerous Conserved Promoters in the Mammalian Genome

**DOI:** 10.1371/journal.pgen.1001134

**Published:** 2010-09-23

**Authors:** Robert S. Illingworth, Ulrike Gruenewald-Schneider, Shaun Webb, Alastair R. W. Kerr, Keith D. James, Daniel J. Turner, Colin Smith, David J. Harrison, Robert Andrews, Adrian P. Bird

**Affiliations:** 1 Wellcome Trust Centre for Cell Biology, University of Edinburgh, Edinburgh, United Kingdom; 2 Wellcome Trust Sanger Institute, Wellcome Trust Genome Campus, Hinxton, Cambridge, United Kingdom; 3 Neuropathology Unit, Division of Pathology, University of Edinburgh, Edinburgh, United Kingdom; 4 Division of Pathology, Institute of Genetics and Molecular Medicine, University of Edinburgh, Western General Hospital, Edinburgh, United Kingdom; The Babraham Institute, United Kingdom

## Abstract

CpG islands (CGIs) are vertebrate genomic landmarks that encompass the promoters of most genes and often lack DNA methylation. Querying their apparent importance, the number of CGIs is reported to vary widely in different species and many do not co-localise with annotated promoters. We set out to quantify the number of CGIs in mouse and human genomes using CXXC Affinity Purification plus deep sequencing (CAP-seq). We also asked whether CGIs not associated with annotated transcripts share properties with those at known promoters. We found that, contrary to previous estimates, CGI abundance in humans and mice is very similar and many are at conserved locations relative to genes. In each species CpG density correlates positively with the degree of H3K4 trimethylation, supporting the hypothesis that these two properties are mechanistically interdependent. Approximately half of mammalian CGIs (>10,000) are “orphans” that are not associated with annotated promoters. Many orphan CGIs show evidence of transcriptional initiation and dynamic expression during development. Unlike CGIs at known promoters, orphan CGIs are frequently subject to DNA methylation during development, and this is accompanied by loss of their active promoter features. In colorectal tumors, however, orphan CGIs are not preferentially methylated, suggesting that cancer does not recapitulate a developmental program. Human and mouse genomes have similar numbers of CGIs, over half of which are remote from known promoters. Orphan CGIs nevertheless have the characteristics of functional promoters, though they are much more likely than promoter CGIs to become methylated during development and hence lose these properties. The data indicate that orphan CGIs correspond to previously undetected promoters whose transcriptional activity may play a functional role during development.

## Author Summary

In the decade since the sequence of the human genome was announced, efforts have been made to annotate all genes with their regulatory sequences. CpG islands are short regions containing the sequence CG at high density that map to regions controlling the expression of most human genes (known as promoters). Using a biochemical method, we have identified and mapped all CpG islands in the human and mouse genomes and find that over half are remote from known gene promoters—so-called “orphans.” Mice, which were thought to possess far fewer CpG islands than humans, turn out to have a very similar number. Surprisingly, orphan CpG islands in both species often mark hitherto unknown promoters. The activity of these novel promoters is particularly dynamic during normal development, as they are often silenced by DNA methylation. In colorectal cancers, however, aberrant DNA methylation affects all CpG islands equally.

## Introduction

In the decade since the human genome sequence was published [1], [2], annotation of its landmarks and functional domains has been a priority. Protein coding genes have been quite comprehensively identified and mapped, but full annotation of the genome is far from complete. In addition to genes, there are DNA sequence categories of likely functional importance, including non-coding transcription units, conserved elements and regions of variant base composition, whose biological significance is not well understood. Into the latter category fall CpG islands (CGIs), which comprise about 1% of the genome and display an elevated G+C base composition spanning approximately 1000 base pairs. Their distinguishing feature is a high frequency of the dinucleotide CpG, but beyond this they do not share long range sequence similarity [3]. In the human genome, CGIs have approximately 1 CpG every 10 base pairs, which is about 10 times more frequent than the surrounding DNA. The high density of CpG shared by CGIs is partly explained by a G+C-rich base composition, but also depends critically on the lack of the CpG deficiency that is typical of the bulk genome. These dense CpG clusters are usually devoid of CpG methylation, whereas the bulk genome is methylated at 70–80% of CpGs. The lack of methylation in the germline [4] means that CGIs do not suffer accelerated mutational loss of CpGs caused by deamination of 5-methylcytosine [5], [6]. Over evolutionary time, this has given rise to the observed contrast between a CpG-deficient bulk genome and relatively CpG-rich CGIs. Clustering of unmethylated CpGs has allowed the CGIs to be biochemically isolated as a relatively homogeneous fraction of DNA [3], [7] or chromatin [8].

CGIs encompass the transcription start site (TSS) of approximately 60% of human protein coding genes. Extensive genome-wide mapping of histone modifications by chromatin immunoprecipitation (ChIP) has established that trimethylation of lysine 4 of histone H3 (H3K4me3) is a signature mark coinciding with most promoter CGIs, even when the associated gene is not expressed [9]–[11]. A potential biological rationalisation for the maintenance of unmethylated CpGs at many promoters has recently emerged from studies of proteins that interact preferentially with CGIs. The protein Cfp1 contains a CXXC domain that specifically binds to CpG only when it is unmethylated and co-localises with almost all CGIs in the mouse genome. Cfp1 is a component of the Set1 complex which trimethylates histone H3 lysine 4 and its depletion drastically affects levels of this modification at CGIs [12]–[14]. Importantly, insertion of a promoterless stretch of CpG-rich DNA into the mouse genome is sufficient to recruit Cfp1 and create a novel peak of H3K4me3 [14]. Complementing this predisposition to form H3K4me3 chromatin is the intrinsic reluctance of CGIs to assemble nucleosomes [15]. Both these features appear to pre-adapt CGIs for active promoter function.

The notion that CGIs facilitate promoter function fits well with their presence at TSSs, but is challenged by two observations that appear to weaken the link with genes. Firstly, genomic analysis has indicated that the number of CGIs in humans and mice is very different, with mice apparently possessing little more than half the number present in humans [16], [17]. Lack of evolutionary conservation would argue against a central role in promoter function. A second reason to query the importance of CGIs has come from the use of CXXC Affinity Purification (CAP) to identify a large fraction of CGIs. Mapping showed that many CGIs in the human genome are not coincident with annotated promoters, but are either intergenic or within the body of coding regions (intragenic) [7]. To clarify these issues we have compiled a comprehensive CGI map for three developmentally distinct human and mouse tissues (sperm, whole blood and cerebellum). The results show that, contrary to previous conclusions, the numbers of CGIs in human and mouse are very similar. Moreover, in both organisms approximately half of all CGIs are remote from annotated promoters. These “orphan” CGIs co-localise with peaks of H3K4me3 and evidence suggests that a large proportion recruit RNA polymerase II (RNAPII) and give rise to novel transcripts. We find that de novo methylation during development predominantly affects orphan CGIs in both humans and mice, with few protein-coding gene promoters being methylated. This contrasts with the situation in colorectal tumors, where cancer-specific de novo methylation affects both CGI categories equally, with a strong preference for those marked in ES (embryonic stem) cells by H3K27me3 – the chromatin modification that is associated with polycomb-mediated repression [18]–[21]. Our findings sustain the notion that all CGIs correspond with promoters and that many orphan CGIs are associated with novel transcripts that may have regulatory significance.

## Results

### Similar abundance and distribution of CpG islands in human and mouse

**Figure 1:**
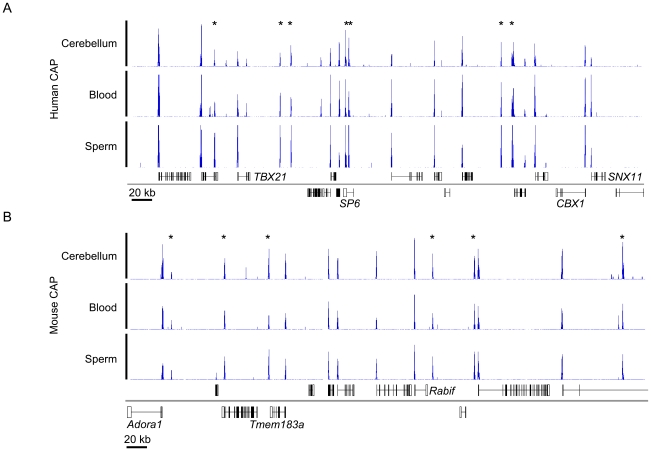
Typical CAP-seq profiles for human and mouse tissues. (A, B) CAP-seq read density profiles (blue) for sperm, blood and cerebellum of human chr17: 43,061,000–43,596,500 (A) and mouse chr1: 136,095,000–136,630,500 (B). Genes (Refseq) are annotated below the CAP-seq profiles with those mapped to the positive and negative strand displayed above and below the chromosome (grey line) respectively. Non-promoter CGIs are denoted by asterisks. See also Figure S1.

CGIs are characteristic of most human and mouse gene promoters, but biochemical and computational studies have suggested that the total number in the mouse genome is over one third less than for human [16], [17]. To check this observation we comprehensively mapped all human and mouse CGIs using CAP to enrich for DNA fragments containing clusters of unmethylated CpGs, in conjunction with high throughput sequencing (CAP-seq) [7]. DNAs from three developmentally distinct tissues, sperm (germline), blood (mesoderm) and cerebellum (ectoderm) were studied. Initial CAP-seq analysis appeared to confirm the lower number of CGIs in mice, but closer examination of syntenic chromosomal regions indicated that the mouse harboured CpG-rich regions that were not efficiently recovered under our CAP conditions (Figure S1A). Bisulfite sequencing established that these regions were in fact unmethylated in the mouse genome and do therefore correspond to potential CGIs (Figure S1B). As the CpG density of the entire mouse CGI set was found to be lower than in human (p-value <2.2×10^−16^; Welch Two Sample t-test; Figure S1C), we concluded that the salt-wash conditions prior to CAP elution were too stringent to allow retrieval of relatively CpG-deficient mouse CGIs. Reduction from 600 mM to 560 mM NaCl corrected this disparity and generated prominent sequence read peaks with minimal intervening background (Figure 1 and Figure S1D). This optimisation resulted in the identification of an additional 7,638 CGIs in mouse which were missed under the more stringent CAP conditions (an increase of ∼50%). Application of the lower stringency wash conditions to CAP of human sperm DNA, however, only identified a further 179 additional CGIs (an increase of 0.7%), indicating that virtually all human CGIs were captured under the previous conditions. (data not shown).

**Figure 2:**
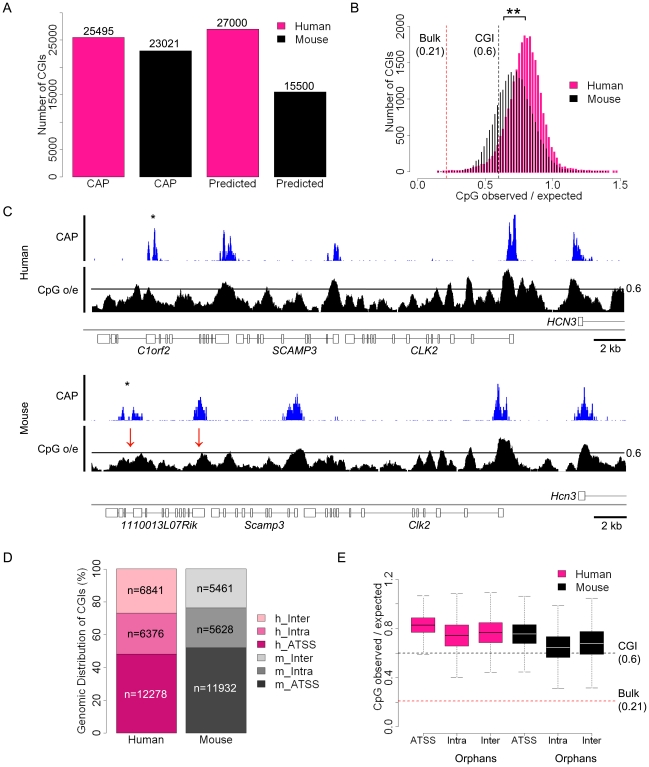
Similar numbers of CGIs in humans and mice, but differing CpG densities. (A) Numbers of human (pink) and mouse (black) CGIs identified by CAP-seq (CAP) and sequence based prediction (Predicted) [17]. Total numbers are noted above each bar. (B) Histogram depicting the CpG observed/expected (o/e) values for all human (pink) and mouse (black) CGIs. Statistical significance (**) was determined using a Welch Two Sample t-Test. The human genome average CpG o/e value of 0.21 (broken red line) and the standard CGI prediction threshold minimum of 0.6 (broken black line) are indicated. (C) CAP-seq (blue) and CpG o/e (black; 400 bp window with a 10 bp slide) profiles for syntenic regions of human (chr16: 16,933,500–17,785,000) and mouse (chr3: 88,960,000–88,995,000) genomes. CGIs missed by standard sequence prediction parameters in mouse are indicated (red arrows). Sequence profiles are displayed as for Figure 1. (D) Categorisation of CGIs with respect to annotated genes (Refseq) in human and mouse. Categories indicated are human and mouse annotated transcription start site associated (h/m-ATSS), human and mouse intragenic (h/m-Intra) and human and mouse intergenic (h/m-Inter). (E) Box plots representing the relative CpG o/e values of CGIs at different genomic locations with respect to genes in human (pink) and mouse (black). Genome average and CGI prediction threshold CpG o/e values are indicated as in (B). CGIs distribution was categorised as either annotated transcription start site (ATSS), intragenic (Intra) or intergenic (Inter). Box plots represent the central 50% of the data (filled box), the median value (central bisecting line) and the whiskers (1.5× the inter-quartile range).

To assess the integrity of the CAP-seq data we compared the average sequence coverage for contiguous 1 kb windows across the whole mouse genome in a panel of CAP purified samples. Figure S2 depicts pairwise comparisons of mouse sperm, blood and cerebellum and includes technical replicates for sperm and cerebellum. The strong correlation between technical replicates indicates that the variance observed between tissues represents *bona fide* biological differences (Figure S2). The similarity of CAP-seq profiles highlights the constitutively hypomethylated state of most CGIs irrespective of the tested tissue (Figure 1A and 1B and Figure S2). By combining regions of substantial CAP-seq enrichment in each tissue (see Materials and Methods) we identified nearly equivalent CGI compliments of 25,495 and 23,021 CGIs in human and mouse, respectively. In the case of human CGIs, these findings are similar to the results from DNA sequence-based prediction methods, which indicated 27,000 CGIs. In mice, however, previous estimates were much lower at 15,500 than those generated by CAP (Figure 2A) [17]. This discrepancy is probably due to the lower average CpG-richness of mouse CGIs compared with human CGIs, as confirmed by CpG density plots (p-value <2.2×10^−16^; Welch Two Sample t-test; Figure 2B). About one fifth of mouse CGIs failed to meet the minimum bioinformatic criterion for CpG density (CpG o/e  = 0.6; dashed black line; [17], although they were significantly more CpG-rich than bulk genomic DNA (CpG o/e in human  = 0.21; dashed red line).

The compositional difference between human and mouse CGIs was apparent when CAP-seq profiles at regions of conserved synteny were compared. As shown in Figure 2C, two mouse CGIs at positions conserved in human and mouse (red arrows) failed to meet the standard sequence criteria employed by most prediction algorithms [17], [22]. It is noteworthy that other regions within these loci approach the CpG density of CGIs, but are not retained by CAP because they are methylated (confirmed by methylation analysis - see below). CAP-seq, which relies on clustering of unmethylated CpGs, is evidently a more accurate assay for CGIs than bioinformatic methods that do not take into account CpG methylation status.

We next determined the location of human and mouse CGIs with respect to annotated genes. Approximately half (12,278 in human and 11,932 in mouse) mapped to annotated gene promoters, the remainder being evenly distributed between intra- and intergenic regions (black asterisks in Figure 2C; see also Figure 1 and Figure 2D). We provisionally refer to CGIs remote from annotated promoters as “orphan-CGIs”, pending a more complete understanding of their roles. Comparison of the sequence composition of annotated promoter CGIs and orphans showed a slightly reduced CpG density in the latter, although the difference compared to bulk genomic DNA remained large (Figure 2E). Taking the data together, we conclude that, while human and mouse CGIs are compositionally distinct, their abundance and genomic distributions are largely equivalent.

### Many orphan CGIs have promoter-like characteristics

**Figure 3:**
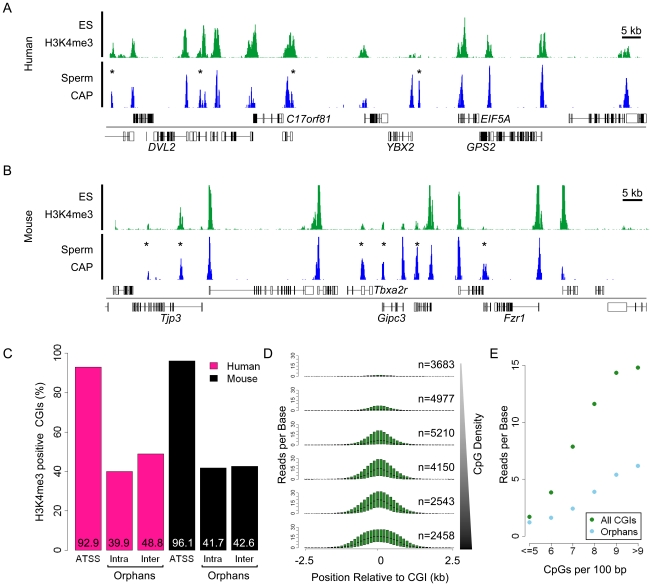
Trimethylated H3K4 is the signature chromatin mark at CGIs and is grossly proportional to CpG density. (A, B) Sequence read profiles for H3K4me3 in ES cells (green) and CAP-seq in sperm (blue) are depicted for human chr17: 7,054,500–7,203,500 (A) and mouse chr10: 80,726,000–80,874,000 (B). (C) Bar plot indicating the percentage (displayed within each bar) of H3K4me3 positive CGIs in human (pink) and mouse (black). Categories of CGI position relative to genes are represented as for Figure 2E. (D) Box plots of H3K4me3 reads per base (averaged across 500 bp with a 100 bp slide) spanning 5 kb surrounding all mouse CGIs at the following CpG densities (CpGs per 100 bp): <5, 5–6, 6–7, 7–8, 8–9 and >9, in ascending order from the top. Box plots represent the distribution of the central 50% of the data (filled box) and the median (black bisecting line). The numbers of islands in each category (n) is noted in parenthesis. Figure S3 shows equivalent data for human CGIs. (E) Summary plot relating the CpG density of each bin to the mean H3K4me3 read value for the central 2 kb of regions displayed in (D). Plots illustrate the relationship for all CGIs (green) and orphan CGIs (blue).

The majority of annotated CGI promoters are highly enriched for both di- and tri-methylated H3K4 in mouse ES cells [11], [23], [24]. To determine if this characteristic is shared by orphan CGIs, we compared the H3K4me3 profile of ES cells with that of CGIs in human and mouse. Sequence reads for both CGIs (blue) and H3K4me3 (green) illustrate the tight association between these marks, including intergenic and intragenic orphan CGIs in each species (Figure 3A and B). To assess this phenomenon globally we intersected CGIs with peaks of H3K4me3 (see Materials and Methods). Consistent with previous reports, over 90% of annotated promoter-associated CGIs in mouse and human ES cells coincide with peaks of H3K4me3 [11], [23]. In addition, about 40% of inter- and intragenic orphan CGIs are associated with H3K4me3 peaks in both species (Figure 3C).

It has been reported that clusters of unmethylated CpG can recruit H3K4me3, a modification associated with sites of transcriptional initiation, via the CpG binding protein Cfp1 [14]. Consistent with these findings we confirmed that the majority of H3K4me3 enriched loci map to CGIs in human and mouse ES cells (74.6 and 84.1% respectively). Based on this result we postulated that the magnitude of H3K4me3 modification may mirror the CpG density at hypomethylated CGIs. Analysis of CGIs, binned according to CpG density, revealed a striking correlation between CpG density and H3K4me3 abundance in mouse and human ES cells (Figures 3D and 3E and Figure S3). The direct relationship between CpG density and H3K4me3 provides support for the notion that CpG plays a causal role in attracting this chromatin mark. Separate analysis of annotated promoter and orphan CGIs confirmed this relationship for both classes (Figure 3E). Visual inspection suggests that many CpG-deficient orphans which score as H3K4me3-negative possess this modification at levels below the detection limit of ChIP-seq.

**Figure 4:**
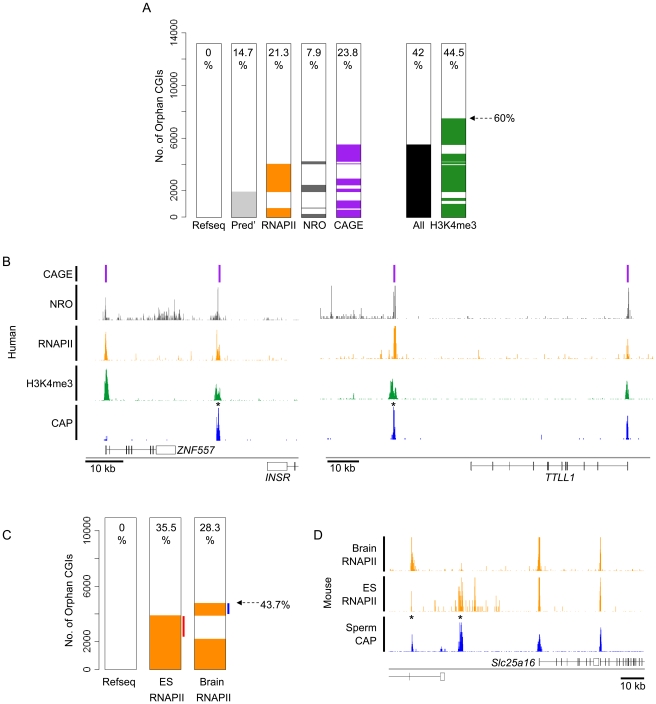
Many orphan CGIs demarcate sites of transcriptional initiation with tissue-restricted activity. (A) A heat map indicating the association of human orphan CGIs (n = 13,217) with predicted gene TSSs (Pred'; grey; data from USCS and Ensembl), RNAPII peaks in human ES cells (orange), nuclear run-on transcripts in human lung fibroblasts (NRO; grey; [34], transcripts detected by multiple tissue Cap Analysis of Gene Expression (CAGE; purple bars; [33]) and H3K4me3 peaks in human ES cells (green). The percentage of overlap is noted within the plot and the complete set of orphan CGIs which overlap a TSS by at least one of the above criteria is indicated (All; black). (B) Examples of orphan CGIs which co-localise with signatures of transcriptional initiation. Mapped sequence reads for Sperm CAP (blue), hES H3K4me3, hES RNAPII, NRO and CAGE are displayed for human chr19: 7,020,000–7,071,000 (left panel) and chr22: 41,721,500–41,819,500 (right panel). Sequence profiles are colour coded as in (A). (C) Heat map depicting the association of RNAPII with orphan CGIs (n = 11,089) in mouse ES cells and brain. Orphan CGIs associated with RNAPII only in ES cells or only in brain are indicated (red and blue lines respectively) and the total percentage expressed is indicated (dashed arrow). (D) Profiles for sperm CAP (blue) and ES cell and brain RNAPII (orange) are depicted for mouse chr10: 62,302,000–62,435,000 showing two orphan CGIs (asterisks) which are differentially associated with RNAPII in mouse ES cells and brain.

Do orphan CGIs represent previously un-annotated promoters? Earlier studies identified unforeseen transcripts originating from orphan CGIs located within the bodies of protein-coding genes [25]–[27]. At a more global level, intergenic sites of H3K4me3 have been linked with conserved ncRNAs [28], [29]. To test whether orphan CGIs mark unanticipated sites of transcriptional initiation we mapped sites of RNAPII recruitment in ES cells using ChIP-seq with an antibody specific for the hypo-phosphorylated (initiating) form [30]. Approximately 21% of human orphan CGIs were associated with RNAPII peaks, pointing to promoter activity in this cell type (Figure 4A). To further test for transcription from orphan CGIs, we compared their localisation with published datasets relating to gene prediction and RNA sequencing. For gene prediction we extracted alternative gene sets hosted by the Ensembl and USCS genome browsers (Figure 4A) [31], [32]. Mapped RNA was assessed using published datasets based on nuclear run on (NRO) and Cap Analysis of Gene Expression (CAGE) [33], [34]. NRO provides a ‘snap shot’ of all nascent RNA in the nucleus by the controlled incorporation of BrU and immunoprecipitation with an antibody against the modified base. NRO-seq in human lung fibroblast cells revealed transcriptional profiles with prominent peaks corresponding to TSSs (as illustrated for *ZNF557* and *TTLL1*; Figure 4B) [34]. CAGE is an independent RNA-based approach which uses methylguanosine cap capture to generate short sequence tags corresponding to the 5′ termini of mature RNAs. CAGE tags generated by deep sequencing of a panel of 12 embryonic and somatic cell types was compared to the human CGI set (Figure 4A) [33]. Each type of dataset implicated a partially overlapping subset of orphan islands in transcription initiation (Figure 4A). Altogether 42% of human orphan CGIs showed evidence for promoter activity by one or more of these criteria. If the presence of a coincident peak of H3K4me3 is included as a marker of promoter function, this proportion increases to 60% (Figure 4A). For example, two intergenic orphan CGIs that are H3K4me3 positive and coincide with sites of RNAPII, NRO and CAGE enrichment are shown in Figure 4B (black asterisks).

To determine if orphan CGIs have tissue specific promoter activity we compared sites of RNAPII occupancy in mouse brain [14] and ES cells. In total, 2,227 (20%) orphan CGIs displayed enrichment for RNAPII in both tissues, with an additional 2,624 (23.7%) being specific to only one tissue (Figure 4C; orphans with tissue specific RNAPII association are indicated by red and blue lines). It is interesting to note that RNAPII occupancy at CGIs associated with annotated promoters display an 87% overlap, whereas coincidence between the two tissues drops to 46% at orphan CGIs. This suggests that orphans display a more tissue restricted expression profile than do annotated promoters as exemplified in Figure 4D. We propose that the absence of promoter signatures at about half of orphan CGIs probably reflects the relatively small number of tissues tested so far. Analysis of additional tissues would likely identify additional novel promoters. Indeed, most or all orphan CGIs may correspond to previously unidentified sites of transcriptional initiation.

### Reciprocal screening identifies preferential methylation of orphan CGIs

**Figure 5:**
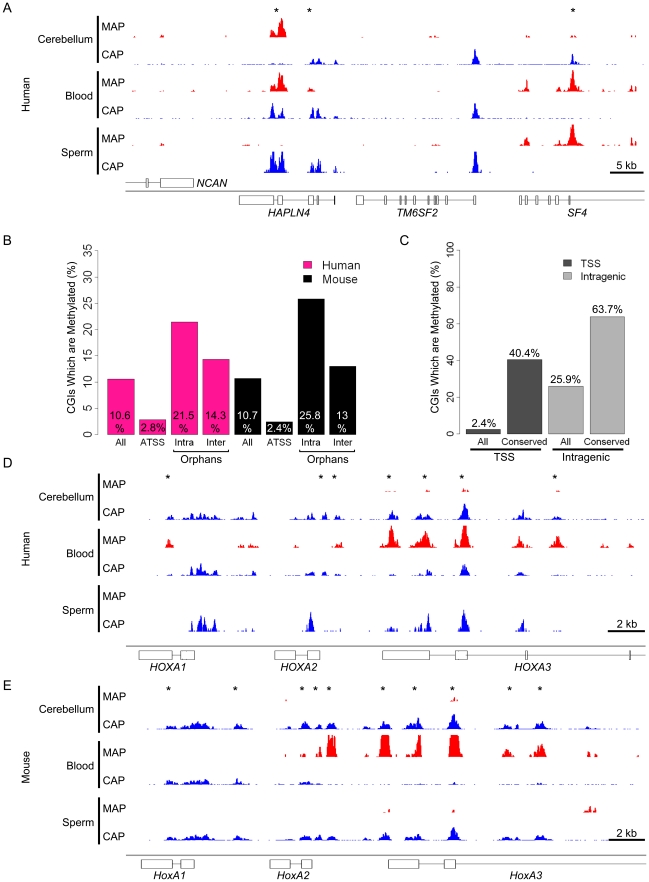
Reciprocal screening identifies inter-species conservation of CGI methylation even at sites distal to annotated promoters. (A) CAP- (blue) and MAP-seq (red) profile for human chr19: 19,218,000–19,264,000. (B) Bar plot representing the percentage of methylated CGIs at different genomic locations for human (pink) and mouse (black). Categories are displayed as in Figure 2E and individual percentages for each are noted within the plot. (C) Preferential methylation at CGIs whose location is evolutionarily conserved between humans and mice. Bar plot depicting the percentage of mouse CGIs which are somatically methylated (All) compared with the percentage of CGIs with identifiable human orthologues (conserved). The percentage of methylation (indicated within the plot) is displayed for CGIs associated with annotated transcriptional start sites (ATSS; black) and orphan CGIs associated with gene bodies (Intragenic; grey). (D, E) Example of conserved orphan CGI methylation in the *HOXA* locus. CAP- (blue) and MAP-seq (red) profiles spanning the first three genes in the *HOXA* locus in human (D) and mouse (E). Regions displayed are human chr7: 27,098,000–27,128,000 and mouse chr6: 52,104,000–52,130,000.

Although the majority of CGIs are unmethylated, a significant fraction becomes methylated in somatic cells [4], [7]. We used MBD (methyl-binding domain) affinity purification (MAP; [3], [7]) to establish the patterns of DNA methylation associated with orphan and annotated promoter CGIs in the same human and mouse tissues that were used for CAP. DNA from each tissue was MAP-selected to enrich DNA sequences with more than 1 methyl-CpG per 100 bp (Figure S4). MAP and CAP data generated reciprocal maps of methylated and unmethylated CGIs respectively. Where MAP-seq signal was high, we observed a reciprocal depletion of the CAP-seq profile as determined by pairwise scatter plots (Figure S5) and illustrated for a CGI located within the *HAPLN4* gene (Figure 5A). Comprehensive genome-wide analysis showed that human and mouse methylate almost identical proportions of CGIs in the two somatic tissues (10.6 and 10.7% respectively; Figure 5B). Both species show a strong preference for methylating orphan CGIs, with intragenic CGIs being even more likely than intergenic CGIs to be methylated (21–26% compared with 13–15%). By contrast, relatively few annotated promoter CGIs become methylated (2.8 and 2.4% in human and mouse respectively), as noted previously (Figure 5B) [7], [35].

To determine if DNA methylation at orphan CGIs has been conserved over evolutionary time between mice and humans, we identified CGIs associated with single-copy orthologous genes and examined their methylation status. We mapped all human methylated CGIs to their orthologous sequences in mouse and determined their methylation status in mouse blood and cerebellum. This analysis showed considerable inter-species conservation as 40% of annotated promoter CGIs and 64% of intragenic orphan CGI were methylated in mice at locations orthologous to the human methylated CGIs (Figure 5C). Intergenic orphans CGIs were not assessed due to difficulties in unambiguously mapping syntenic regions lacking annotated genes. The results demonstrate conservation of CGI methylation even at sites distal to gene promoters. For example, Figure 5D and E shows a cluster of orphan CGIs located within the *HOXA* locus that exhibits a closely related pattern of blood-specific DNA methylation in each species.

**Figure 6:**
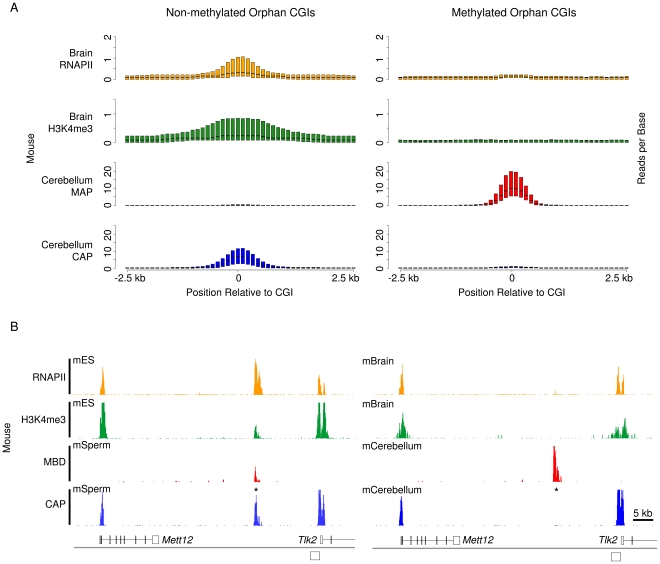
Somatic methylation is incompatible with H3K4me3 and RNAPII occupancy at orphan CGIs. (A) Composite box plots showing sequence read density for brain RNAPII (orange), brain H3K4me3 (green), cerebellum MAP (red) and cerebellum CAP (blue) in mouse. Plotted as for Figure 3D. (B) Sequence profiles of CAP, MAP, H3K4me3 and RNAPII for mouse (chr11: 104,982,000–105,056,000) in ES cells and sperm (left panel) and brain and cerebellum (right panel) depict the loss of RNAPII and H3K4me3 associated with a gain of DNA methylation in cerebellum. Sequence profiles are colour coded as for (A).

There is extensive evidence that CGI methylation inhibits transcriptional activity when coincident with gene promoters [36], [37]. To determine the effect of methylation at orphan CGIs, we compared published H3K4me3 and RNAPII ChIP-seq data from mouse brain [14] with methylated and unmethylated sets. Unmethylated orphan CGIs were associated with both H3K4me3 and RNAPII, whereas methylated CGIs detected by MAP lacked both H3K4me3 and RNAPII (Figure 6A). As CGIs are generally unmethylated in both sperm and ES cells [4], [24], we compared sperm CAP and MAP profiles with data from ES cell chromatin. An orphan CGI downstream of the *Mett12* gene was shown to be associated with RNAPII and H3K4me3 in ES cells but not in brain where the orphan CGI is heavily methylated (Figure 6B; black asterisk). Given the frequent association between orphan CGIs and novel TSSs, these data indicate that DNA methylation correlates with transcriptional silencing at these loci.

### Orphan CGIs are not preferentially methylated in colorectal carcinomas

**Figure 7:**
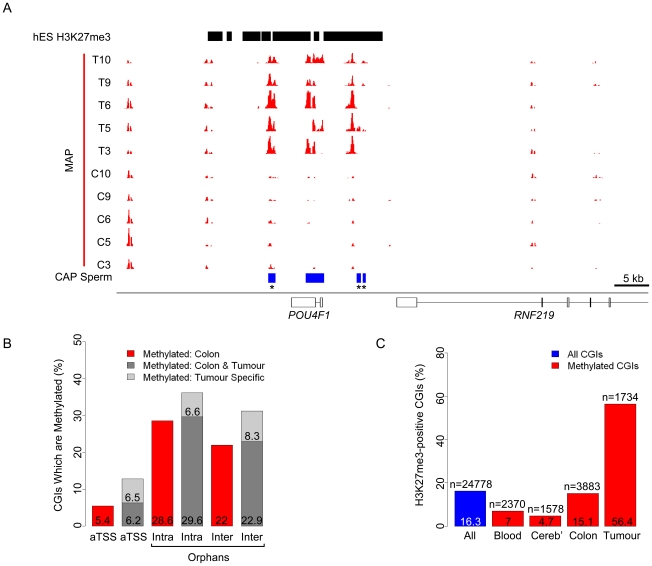
Distinct characteristics of normal and tumour-specific CGI methylation. (A) MAP-seq profiles (red) for five colon mucosa (C3, C5, C6, C9 and C10) and five matched colorectal tumour (T3, T5, T6, T9 and T10) biopsy samples corresponding to human chr13: 78,052,000–78,123,500. CGIs (blue bars) and sites of hES H3K27 trimethylation (hES H3K27me3; black bars; [46] are represented). See also Figure S7. (B) Bar plot representing the percentage of autosomal CGI methylation in colon (red bars), colon and tumour (dark grey) and tumour only (light grey) relative to gene position (categorised as for Figure 2E). (C) Bar plot indicating the percentage of all autosomal (blue) and methylated autosomal CGIs (red) which co-localise with domains of H3K27me3 in human ES cells. Percentages and number of CGIs (n) are displayed within the plot. Tumour specific denotes CGIs that are methylated in at least three colorectal carcinoma samples, but not in any of the normal colon samples.

Silencing of tumour suppressor genes by unscheduled *de novo* methylation of their promoter CGIs has been proposed as a primary event leading to unchecked proliferation in neoplastic cells [38]–[41]. Do the sites methylated in cancer represent disease-specific events or do they recapitulate CGI methylation seen during normal development [39]? To address this question, we performed MAP-seq on DNA from ten primary biopsy samples comprising matched colon mucosa (C3,C5,C6,C9 and C10) and colorectal cancer tumors (T3,T5,T6,T9 and T10). MAP-seq profiles identified 1,734 CGIs which were heavily methylated in at least three of the five cancer biopsies but hypomethylated in all normal mucosal samples. Separating out cancer-specific from mucosal CGI methylation events, it was apparent that many of the former were shared by all tumors (39%). This finding highlights the relative homogeneity of CGI methylation in different colorectal tumors (Figure S6). Examples at the *POU4F1* and *PDX1* genes are shown (Figure 7A and Figure S7; CGIs represented as blue boxes). Tumors largely preserved the CGI methylation profile seen in normal mucosa, but additional tumour-specific CGI methylation differed from that in normal tissues by not being preferentially targeted to orphans (Figure 7B). In fact the proportion of intergenic, intragenic and annotated promoter CGIs in the tumour-specific category were approximately equal (Figure 7B). Therefore the novel CGI methylation events accompanying cancer do not recapitulate those seen during normal development.

It has been reported that CGIs that are aberrantly methylated in neoplastic cells coincide with sites targeted by polycomb in human ES cells [42]–[45]. We asked if the distribution of CGI methylation in the colonic mucosa and colorectal tumour samples reflects differential association with histone H3K27 trimethylation in ES cells, a modification deposited by the Polycomb Repressive Complex 2 (PRC2) [18]–[21]. Approximately 16% of all CGIs are H3K27me3 positive in human ES cells [46] and these sites are not over-represented among the CGIs methylated in blood, cerebellum or normal colon (Figure 7C). On the other hand, 56% of tumour-specifically methylated CGIs are derived from CGIs that were H3K27 trimethylated in embryonic cells (Figure 7C). Examples of embryonic H3K27me3 domains that give rise to methylated CGIs in tumors are shown in Figures 7A and Figure S7. These findings emphasise the distinction between tumour-specific CGI methylation and that found in normally developing human somatic tissues and reinforce the link between polycomb domains and tumour-specific CGI methylation. The proportions of orphan and annotated promoter CGIs that are H3K27me3-associated in ES cells is relatively similar (20% and 14% respectively), accounting for the comparable numbers of tumour-specifically methylated CGIs in each category.

## Discussion

### Equivalent CpG island complements in human and mouse

In order to detect all CGIs in human and mouse tissues, we used CXXC-affinity purification in combination with deep sequencing to identify clusters of unmethylated CpG (CAP-seq). This protocol has the advantage that fluctuations in base composition that can erroneously register as CGIs using algorithmic methods are not recovered by CAP unless they are free of CpG methylation. As the vast majority (∼99%) of genomic DNA is both CpG-deficient and heavily methylated (70–80%), false-positive identification of CGIs is minimal. The absence of significant background signal in CAP-seq profiles verifies that CGIs represent a discrete fraction of the mammalian genome that is conserved during evolution. Computational prediction methods suggest that the human genome contains almost twice as many CGIs as mouse [17], but this difference reflects a bias of sequence-based prediction methods due to the somewhat lower average CpG density of mouse CGIs. In fact humans and mice have similar numbers of CGIs (25,500 and 23,000 respectively). We found that an almost identical proportion of gene promoters are embedded within CGIs in each species (59% in human and 60% in mouse).

Although broadly similar, the number of CGIs in humans exceeds that in mouse by ∼2,500. Specific examples contributing to this difference are known; for example the human *α-globin* gene is CGI-associated, whereas this is replaced in mouse by a methylated CpG-deficient promoter [47]. A recent study identified a novel orphan CGI embedded within the transcription unit of human *Retinoblastoma* (*RB1*) gene. This CGI, which is absent in mouse, is an imprinted promoter that gives rise to a transcript regulating *RB1* transcription in cis [48]. Therefore despite the relative similarity between the CGI compliments in human and mouse there remain functionally important differences.

### Active chromatin and transcriptional initiation at most CpG islands

More than half of all CGIs in human and mouse (more than 10,000) can be classed as orphans that are remote from annotated promoters, being either embedded within coding regions or between transcription units. Trimethylation of H3K4, a signature chromatin modification associated with sites of transcriptional initiation [10], is present at over 40% of these unattached CGIs in mouse ES cells, raising the possibility that many are unknown promoters. Interestingly, the magnitude of H3K4me3 modification at CGIs is directly correlated with the density of hypomethylated CpG clustering. This is true at both annotated promoter CGIs and orphans and is consistent with the observation that artificial clusters of unmethylated CpG are sufficient to generate novel peaks of H3K4me3 in transgenic ES cells [14].

There is evidence that CGIs are intrinsically poor substrates for nucleosome assembly and therefore have a reduced requirement for chromatin remodelling to induce transcription [15]. Given the frequent association with H3K4me3 even at orphan CGIs [14], it seems possible that all CGIs might provide a platform on which transcriptional initiation can occur. Indeed many non-coding transcripts, including *Xist*, *Tsix*, *Air* and *HOTAIR*, are transcribed from CGIs embedded within or between the coding regions of other genes [49]–[51]. Additionally, several thousand conserved non-coding RNAs were identified by assessing sites of H3K4me3 juxtaposed to extended domains of H3K36me3 [28], [29]. By combining data from gene prediction annotations, sites of RNAPII occupancy and nascent transcript mapping, we confirmed that 5548 (42%) and 4851 (44%) of orphan CGIs co-localised to sites of transcriptional initiation in human and mouse respectively. The different methods produced overlapping sets, but each also revealed novel orphan promoters not seen by other methods (Figure 4A). This variability may reflect differences in technical sensitivity, RNA turnover or different CGI promoter usage between cell types as illustrated in Figures 4C and 4D. In support of the latter interpretation is the finding that many orphan CGIs are positive for RNAPII and H3K4me3 in brain but not ES cells (912 and 1046 respectively). Pending a comprehensive transcriptional analysis of numerous embryonic and somatic tissues, it is reasonable to assume that 42 and 44% are minimum estimates of the number of orphan CGIs with promoter activity in human and mouse. Based on the data so far, we propose that most or all orphan CGIs correspond to promoters with tissue-restricted patterns of expression. It is likely that the products of orphan CGI promoters are non-coding RNAs, though some may represent alternative transcription start sites of protein-coding genes. We considered the possibility that orphan CGIs include transcribed enhancer elements of the kind recently identified by Kim et al (2010). This seems unlikely, however, as only 440 out of 28,004 enhancers (1.6%) map within 100 bp of orphan CGIs (data not shown).

### CGI methylation is conserved even at sites distal to annotated gene promoters

Orphan CGIs resemble annotated promoter CGIs in their sequence properties, promoter-like chromatin state and general lack of DNA methylation. Their enhanced propensity to become methylated during development, however, is distinctive. To visualise CGI methylation, we applied the reciprocal technologies of CXXC and MBD affinity purification to DNA from human and mouse tissues. Using this positive-negative screen, we established that both human and mouse methylated about 11% of all CGIs in the somatic cell types that were tested. Strikingly, the great majority of CGI methylation events occurring in these normal tissues involve orphan CGIs. Annotated promoter CGIs, by contrast, are infrequently methylated during development. Among orphans, we found that intragenic CGIs were almost twice as likely as intergenic CGIs to become methylated. An intriguing possibility is that many intragenic orphans represent alternative promoters which are utilised in a spatially or temporally restricted fashion, as described for *Pax6* [26]. The high frequency of methylation at this sub-class of orphan CGIs may serve to regulate the expression of such alternative transcripts. The functional significance of the resulting transcripts is a matter of speculation, but one attractive possibility is that they encode non-coding RNAs that are involved in regulation of protein coding gene expression. In this case, highly regulated expression patterns may be required to facilitate tissue-specific programmes of gene expression.

Conservation of methylation patterns at orphan CGIs in humans and mice, which diverged from a common ancestor about 75 million years ago, suggests a functional role for these putative promoters. The phenomenon is illustrated by the *HOXA* locus where a conserved domain of orphan CGIs showed blood-specific methylation in both species. This aligns with previous suggestions that developmental genes, typified by *HOX*, are enriched for methylated CGIs [7]. The functional role of CGI methylation at these sites remains unclear. If all CGIs represent transcription start sites, it is conceivable that methylation at these sites may serve to repress regulatory non-coding RNAs such as *HOTAIR* [49]. Consistent with this hypothesis we found that somatic acquisition of DNA methylation correlates with a precipitous depletion of RNAPII and the active histone modification H3K4me3 at orphan CGIs. This suggests that orphan CGI promoters are regulated by DNA methylation in the same manner as for annotated CGI promoters.

### Sites of CGI methylation distinguish normal and neoplastic colon cells

In cancer, aberrant silencing of tumour suppressor genes often coincides with the abnormal acquisition of CGI methylation [38], [39], [41], [42], [45]. It has been proposed that such abnormal CGI methylation is instructed by polycomb silencing, as sites of DNA methylation in tumors are frequently trimethylated at H3K27 in ES cells [42], [43], [45]. In strong support of this link, we observed a three-fold over-representation of H3K27me3-associated CGIs among the tumour-specifically methylated CGI complement of malignant colorectal cancers. A link that might predispose sites of H3K27me3 to DNA methylation has been proposed [52] yet the mechanistic interplay between these repressive systems remains uncertain, as polycomb repression and CGI methylation appear to be alternative silencing mechanisms in ES cells, with little target site overlap [23], [24]. Recent evidence, however, suggests that breast cancer cells possess an altered PRC2 binding pattern reminiscent of embryonic fibroblasts [53]. If this disrupted chromatin pattern is typical of all neoplastic cells it could facilitate direct crosstalk between sites of H3K27me3 and the DNA methylation machinery.

Colorectal tumour-specific CGI methylation affects annotated promoter and orphan CGIs equally. In contrast, normal colon showed a typical somatic distribution of methylated CGIs as seen in blood and cerebellum, whereby orphan CGIs were preferentially methylated (Figure 7B). Interestingly, there was no discernable preference for methylation of H3K27me3 sites in these normal tissues. We conclude that abnormal CGI methylation that has arisen in cancer is distinct from that which occurs during development. It has been proposed that DNA methylation serves to lock in a pseudo-pluripotent state via acquisition of DNA methylation at CGIs subject to embryonic polycomb repression, thereby facilitating cellular proliferation in neoplastic cells [42], [43], [45]. This scenario involves methylation of CGIs not normally regulated by this mechanism and it highlights the distinction between developmental CGI methylation and that associated with cancer.

### Concluding remarks

Our results establish that CGIs represent a distinctive fraction of the mammalian genome that is conserved between humans and mice. The relationship between CpG density and degrees of H3K4me3 supports a role for CpG in signalling a “promoter-friendly” chromatin conformation. Although about half of CGIs in both species are not previously annotated, our data suggests that these nevertheless represent thousands of functional promoters, often as novel genes for non-coding RNAs. In terms of transcription and DNA methylation, expression of orphan CGI promoters is more highly developmentally regulated than CGIs at annotated protein-coding genes. This observation raises the possibility that the resulting transcripts play functionally important roles during development.

## Materials and Methods

### Ethics statement

Work involving the use of human post-mortem brain samples was approved by the Lothian Research Ethical Committee (Ref. 2003/8/37). All donors consented to the use of this material for DNA extraction and all samples were anonymized prior to DNA extraction. DNA from normal and neoplastic colon tissues were prepared under ethics 08/S1101/41 through the Edinburgh Experimental Cancer Medicine Centre. All mouse work was carried out in accordance with Home Office regulations. No liscenced procedures were required for this work.

### Preparation of human and mouse tissues

Human whole semen (n = 3; aged 24, 26 and 61), whole male blood (n = 3; aged 24, 26 and 61) and whole female blood (n = 5) was collected from healthy donors. Human semen was centrifuged at 5000 g for 5 min then washed three times in PBS to yield pure sperm. All donors consented to the use of this material for DNA extraction and all samples were anonymized prior to DNA extraction. Approximately 500 mg of human cerebellum was provided for three males (aged 41, 50 and 54) and three females (aged 44, 49 and 51) by the MRC Sudden Death Brain Bank, Edinburgh.

Mouse blood was extracted from male (n = 9; aged 30 weeks) and female (n = 8; aged 15 weeks) wild type C_57_Bl/6J mice. Testis (n = 4) and cerebella (n = 3 for male and female) were dissected from a subset of mice used for blood extraction. Testes were dounce homogenised on ice in 310 mM sucrose, 3 mM MgCl2, 10 mM potassium phospahate (pH 6) and 0.05% v/v Triton X-100 and cells were then centrifuged at 800 g for 20 min at 4°C. Cells were resuspended in water and subjected to 3×10 bursts of sonication on ice at setting 2 with a duty cycle of 20% using a Branson digital sonifer. Sonicated cells were then pelleted twice through a 2 ml 1.5 M sucrose cushion at 1000 g for 30 min at 4°C. Pelleted cells were visually inspected to ensure the presence of pure sperm heads.

### DNA extraction

All human and mouse tissues were pooled prior to DNA extraction. DNA was extracted from 10 mls of whole blood using the Genomic-tip 500/G kit according to manufacturers instructions (Qiagen; 10262). Purified sperm cells were incubated in 6 M guanidinium hydrochloride, 30 mM sodium citrate, 0.5% w/v sarkosyl, 0.2 mg/ml proteinase K, 0.2 mg/ml RNase A and 0.3 M β-mercaptoethanol and incubated at 55°C for 4 hours. DNA was isopropanol precipitated as previously described [54]. 300 mg of frozen, cerebella were ground into a fine powder and lysed as for sperm. Lysed material was then extracted once with phenol:chloroform:isoamyl alcohol and once with isoamyl alcohol:chloroform and phased using MaxTract High density columns according to manufacturers instructions (Qiagen; 129065). DNA was then extracted from the aqueous phase by the addition of 5 volumes of isopropanol. All DNA was resuspended in 1x TE buffer.

### Cultured cells

Human Shef 4 ES cells [55] were a gift from Dr Andrew Smith (Institute for Stem Cell Research, University of Edinburgh). Mouse ES cells (E14 TG2a) were grown as previously described [56].

### Bisulfite sequencing

Bisulfite sequencing was performed as previously described [7]. DNA was sonicated using a Diagenode Bioruptor for 10 seconds on high setting prior to bisulfite treatment.

### DNA chromatography: MAP and CAP

MAP was performed using two sequential rounds of chromatography as previously described [57]. For CAP, DNA was prepared as for MAP-seq and recombinant CXXC was expressed and purified as previously described [7]. To remove bacterial DNA, 100 mg of purified CXXC was incubated at room temperature for 90 min with 700 units of DNaseI supplemented with 1x DNaseI reaction buffer (Fermentas; EN0521). CXXC was bound to nickel charged sepharose beads at 50 mg/ml bead volume (GE Healthcare; 17-0575-01). DNA was bound to the CXXC matrix in 0.1 M NaCl containing column buffer, washed at 600 mM NaCl or 560 mM NaCl and then eluted using buffer containing 1 M NaCl. CAP was performed once per sample as this generated sufficient enrichment for Solexa sequencing. Eluted fractions were pooled, concentrated and precipitated as for MAP [57]. A minimum of two independent technical replicates were performed for each DNA sample.

### Chromatin immunoprecipitation (ChIP)

ChIP was performed on human and mouse ES cells as previously described [58]. For each immunoprecipitation, cross-linked chromatin from approximately 10 million cells was incubated with either 1.5 µg of anti-H3K4me3 (ab8580; Abcam) or 5 µg of anti-RNA Polymerase II (ab817; Abcam) antibodies for 16 hrs at 4°C. For each immunoprecipitation two independent replicates were performed.

### Library preparation and Illumina Solexa sequencing

MAP and CAP input DNA was ligated to solexa sequencing adaptors prior to purification as previously described for MAP [57]. CAP, MAP and ChIP solexa libraries were prepared as previously described [57]. Solexa sequencing was carried out at the Wellcome Trust Sanger Institute. Single end reads were mapped to human and mouse reference genome builds (hg18 - NCBI36 and mm9 - NCBIm37 respectively) using MAQ (http://maq.sourceforge.net/). Reads with a mapping score greater or equal to 30 where retained.

### Analysis of high-throughput sequence data

Mapped solexa sequence in the form of. WIG files was processed and analysed using a set of novel tools based on R and perl scripts interfaced with the Galaxy server (http://main.g2.bx.psu.edu/) [59]. Individual replicate solexa lanes were visually inspected using the interactive genome browser [60] and combined to generate single datasets for each biological sample. An overview of all solexa sequencing data is provided in Table S1. The parameters applied in each analysis step are outlined in Table S1. They are; read height (H), length in bp (L) and gap permitted in the length parameter (G). The gap parameter allows for small interruptions in regions which are otherwise represented by contiguous sequence reads.

#### Data normalization

Raw sequencing data was normalised in order to make samples from the same purification directly comparable. Like samples (i.e. all CAP) were scaled to a constant approximating the “average” read number for that purification in order to account for variable sequence depth. In the case of MAP samples, background was removed prior to normalisation as background reads could skew the normalisation procedure. Parameters applied for normalisation are outlined in Table S1.

#### Peak-finding

Peaks of enrichment were identified using H, L and G parameters as outlined in Table S1. Peak-finding was calibrated for each purification to identify regions of known DNA methylation or histone modification state. CAP-seq peak-finding was tailored to identify hypomethylated CGIs previously identified, however the high signal-to-noise ratio obtained meant that varying these parameters did not greatly alter the efficiency of CGI identification. Conversely, MAP-seq peak finding was calibrated to identify well characterised methylated CGIs such as those on the inactive X chromosome in females. For each data type the parameters were kept consistent between all like sample for both species.

Regions of CAP-seq enrichment in sperm, blood and cerebellum were combined to give a comprehensive set of CGIs for both human and mouse. For mouse CGIs, CAP-seq enriched regions identified at both high and low stringency (see above) were combined to account for the relative CpG deficiency of mouse CGIs (the subset of CGIs only identified under stringent conditions are outlined in Dataset S2). CGIs +/−100 bp were intersected with additional genomic features such as predicted genes, domains of H3K4me3 and RNAPII binding. Where required, published datasets were converted to mm9 and hg18 using the liftover tool in galaxy [59]. CGIs were classified as promoter associated, intra- and intergenic based on their overlap +/−100 bp with Refseq annotated genes (33,258 and 25,767 in Human and Mouse respectively). CGIs were designated as promoter associated if they overlapped the 5′ end of an annotated gene (+/−100 bp).

#### Global analysis of sequence data

To determine the relationship between technical replicates and experimental variables, the mean read depth was calculated for every contiguous 1 kb window in the human and mouse genomes. Pair-wise plots were generated for each comparison of interest and the correlation was assessed by calculating the Pearson correlation coefficient using the ‘cor’ function in R.

#### Identification of differentially methylated CGIs

To accurately identify differentially methylated CGIs, a sensitive “sliding window” analysis was carried out on MAP-Seq samples. For each CGI the average number of reads per base was calculated for a 100 bp window with a 20 bp slide. Values for each window were then compared between sperm (hypomethylated reference) and each of the somatic tissue samples. This gave a ratio for each window. If both windows being compared contained less than 4 reads this ratio was set to 1 in order to remove bias due to small fluctuations at low read depth.

Differentially methylated CGIs were defined as those containing 9 out of 10 contiguous windows with a log2 ratio of >2. CGIs were then scored as −1 (less methylated than sperm), 0 (same methylation as sperm) and 1 (more methylated than sperm). These parameters were verified using CGIs on the X chromosome which are methylated specifically in females.

All analytical results are summarised in Datasets S1 and S2 for human and mouse respectively. High-throughput sequencing data have been deposited in the Gene Expression Omnibus (GEO) under the accession number: GSE21442.

## Supporting Information

Dataset S1 Summary of Human CGI data. Summary of all analysed data with respect to Human CGIs.
(8.56 MB XLS)

Dataset S2 Summary of Mouse CGI data. Summary of all analysed data with respect to Mouse CGIs.
(6.47 MB XLS)

Figure S1 Preliminary characterisation of Human and Mouse CAP-seq results. (A) Sperm CAP-seq read density profiles (blue) for human and mouse sperm generated by washing DNA bound to the CXXC column with 600 mM NaCl prior to elution. CpG density (black; 300 bp windows with a 10 bp slide) at 4 human and mouse syntenic chromosomal locations is shown below the read profiles. Genes (Refseq) are annotated below the CAP-seq profiles with those mapped to the positive and negative strand displayed above and below the chromosome (grey line) respectively. The CpG density of 5 CpGs per 100 bp (dashed black line) is indicated for reference. Mouse regions assessed by bisulfite sequencing are indicated (bisulfite; grey bars). (B) Bisulfite sequencing of four putative mouse CGI island promoters. Open circles represent unmethylated CpG sites. Each column represents a single PCR amplicon and horizontal lines represent single sequenced DNA clones. Vertical strokes represent the relative CpG position within each amplicon. (C) Histogram depicting the CpG observed/expected (o/e) values for all human (pink) and mouse (black) CGIs identified by CAP with washing at 600 mM NaCl. Statistical significance (**) was determined using a Welch Two Sample t-Test and CpG o/e values of 0.21 (broken red line; human genome average) and 0.6 (broken black line; standard CGI prediction parameter) are indicated. (D) Sperm CAP-seq read density profiles (blue) for mouse sperm generated by washing with the optimised NaCl concentration (560 mM) in comparison with CpG density (black; 300 bp windows with a 10 bp slide).
(0.19 MB PDF)

Figure S2 Pairwise analysis of mouse CAP-seq data. Scatter plots of CAP-seq data representing the mean sequence read depth for every contiguous 1 kb window in the mouse genome. Each pairwise comparison was assessed by calculating a Pearson correlation coefficient, which is presented above each plot. Tissue and replicate status for pairwise comparisons are noted above and to the left of the plots.
(1.26 MB PDF)

Figure S3 Proportional relationship between CpG density and H3K4me3 at Human CGIs. Box plots of H3K4me3 reads per base (averaged across 500 bp with a 100 bp slide) spanning 5 kb of all human CGIs at different CpG densities (CpGs per 100 bp). CpG density categories applied are ≤5, 5–6, 6–7, 7–8, 8–9 and >9 CpGs per 100 bp, arranged in ascending order from top to bottom. Box plots represent the distribution of the central 50% of the data (filled box) and the median (black bisecting line). The numbers of islands in each category (n) is noted in parenthesis.
(0.04 MB PDF)

Figure S4 Characterisation of MAP enrichment. Histograms representing the CpG density of MAP-enriched genomic loci in human (hMAP) and mouse (mMAP). The vertical dashed red line represents the lower tenth percentile of the data indicating that the majority of characterised MAP enriched DNA fragments have a CpG density of at least 1 and 1.3 CpGs per 100 bp in human and mouse respectively.
(0.05 MB PDF)

Figure S5 Global scatter plots reveal a reciprocal relationship between CAP- and MAP-seq data for human sperm, blood, and cerebellum. Scatter plots display pairwise comparisons of CAP- and MAP-seq data for every contiguous 1 kb window in the human genome using normalised data for human sperm, blood and cerebellum. Plots are represented as for Figure S2.
(0.12 MB PDF)

Figure S6 Pairwise comparisons of MAP-seq data reveal consistent tumour-specific methylation. Scatter plots displaying pairwise comparisons of MAP-seq data for every colon (C) and colorectal tumour (T) sample screened by MAP-seq. Data represents the mean sequence depth for every 1 kb window in the human genome. Data is presented as for Figure S2.
(4.94 MB PDF)

Figure S7 Tumour-specific CGI methylation associated with *PDX1*. MAP-seq profiles (red) for five colon mucosa (C3, C5, C6, C9 and C10) and five matched colorectal tumour (T3, T5, T6, T9 and T10) biopsy samples for human chr13: 27,325,000–27,402,000. CGIs (blue bars) and sites of hES H3K27 trimethylation (hES H3K27me3; black bars; [46] are represented).
(0.05 MB PDF)

Table S1 Sample information and data analysis parameters. Summary of the sequence and analysis statistics for each biological sample presented.
(0.04 MB XLS)
