# Supplementary figures and images for: Orphan CpG Islands Identify Numerous Conserved Promoters in the Mammalian Genome
Source: PLoS Genet. 2010 Sep 23;6(9):e1001134. doi: 10.1371/journal.pgen.1001134 (PMC2944787; doi:10.1371/journal.pgen.1001134)

# Supplementary Figure 1

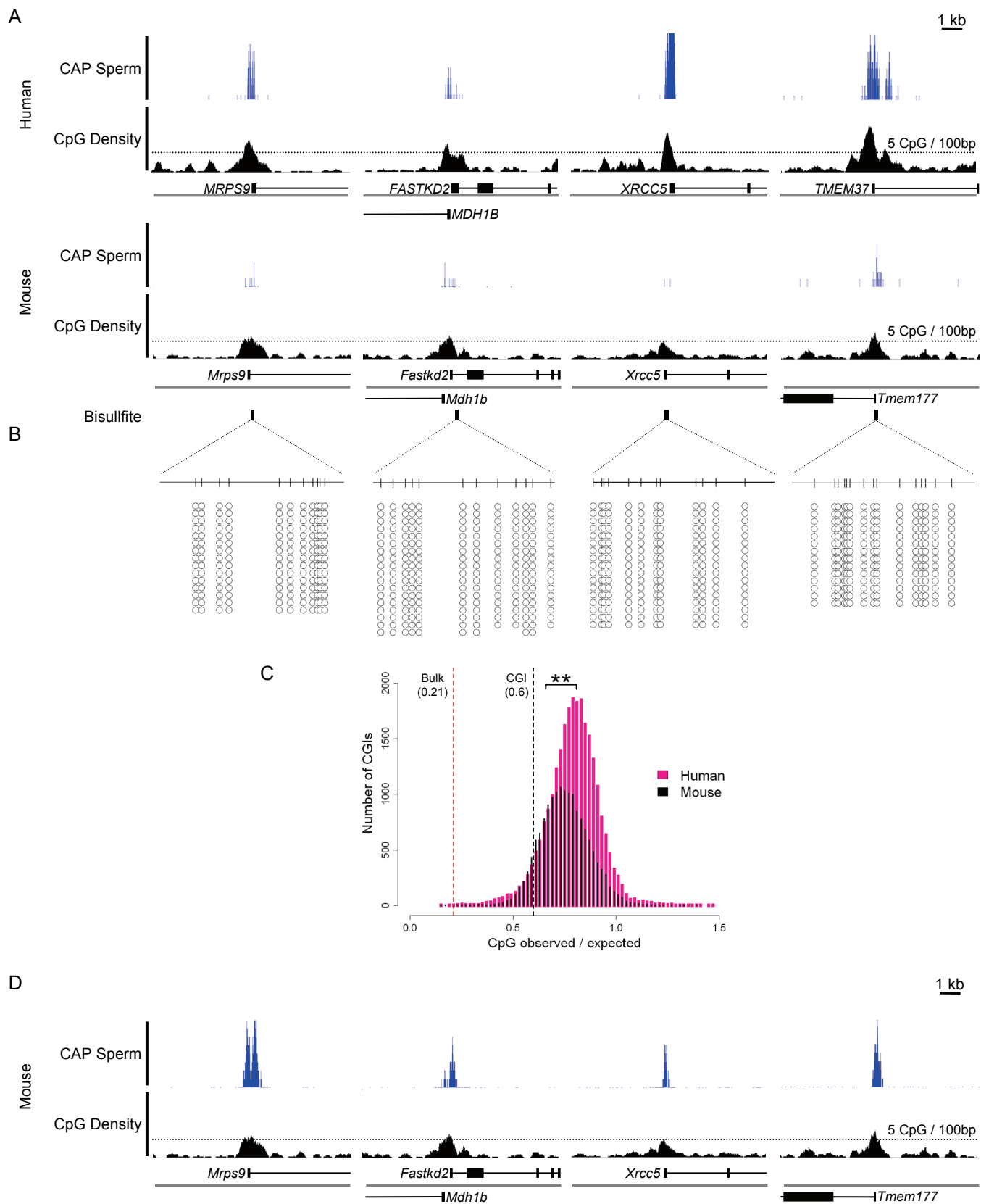

Supplement: Figure S1 — Preliminary characterisation of Human and Mouse CAP-seq results. (A) Sperm CAP-seq read density profiles (blue) for human and mouse sperm generated by washing DNA bound to the CXXC column with 600 mM NaCl prior to elution. CpG density (black; 300 bp windows with a 10 bp slide) at 4 human and mouse syntenic chromosomal locations is shown below the read profiles. Genes (Refseq) are annotated below the CAP-seq profiles with those mapped to the positive and negative strand displayed above and below the chromosome (grey line) respectively. The CpG density of 5 CpGs per 100 bp (dashed black line) is indicated for reference. Mouse regions assessed by bisulfite sequencing are indicated (bisulfite; grey bars). (B) Bisulfite sequencing of four putative mouse CGI island promoters. Open circles represent unmethylated CpG sites. Each column represents a single PCR amplicon and horizontal lines represent single sequenced DNA clones. Vertical strokes represent the relative CpG position within each amplicon. (C) Histogram depicting the CpG observed/expected (o/e) values for all human (pink) and mouse (black) CGIs identified by CAP with washing at 600 mM NaCl. Statistical significance (**) was determined using a Welch Two Sample t-Test and CpG o/e values of 0.21 (broken red line; human genome average) and 0.6 (broken black line; standard CGI prediction parameter) are indicated. (D) Sperm CAP-seq read density profiles (blue) for mouse sperm generated by washing with the optimised NaCl concentration (560 mM) in comparison with CpG density (black; 300 bp windows with a 10 bp slide). (0.19 MB PDF) [file pgen.1001134.s003.pdf]

Supplementary Figure 2

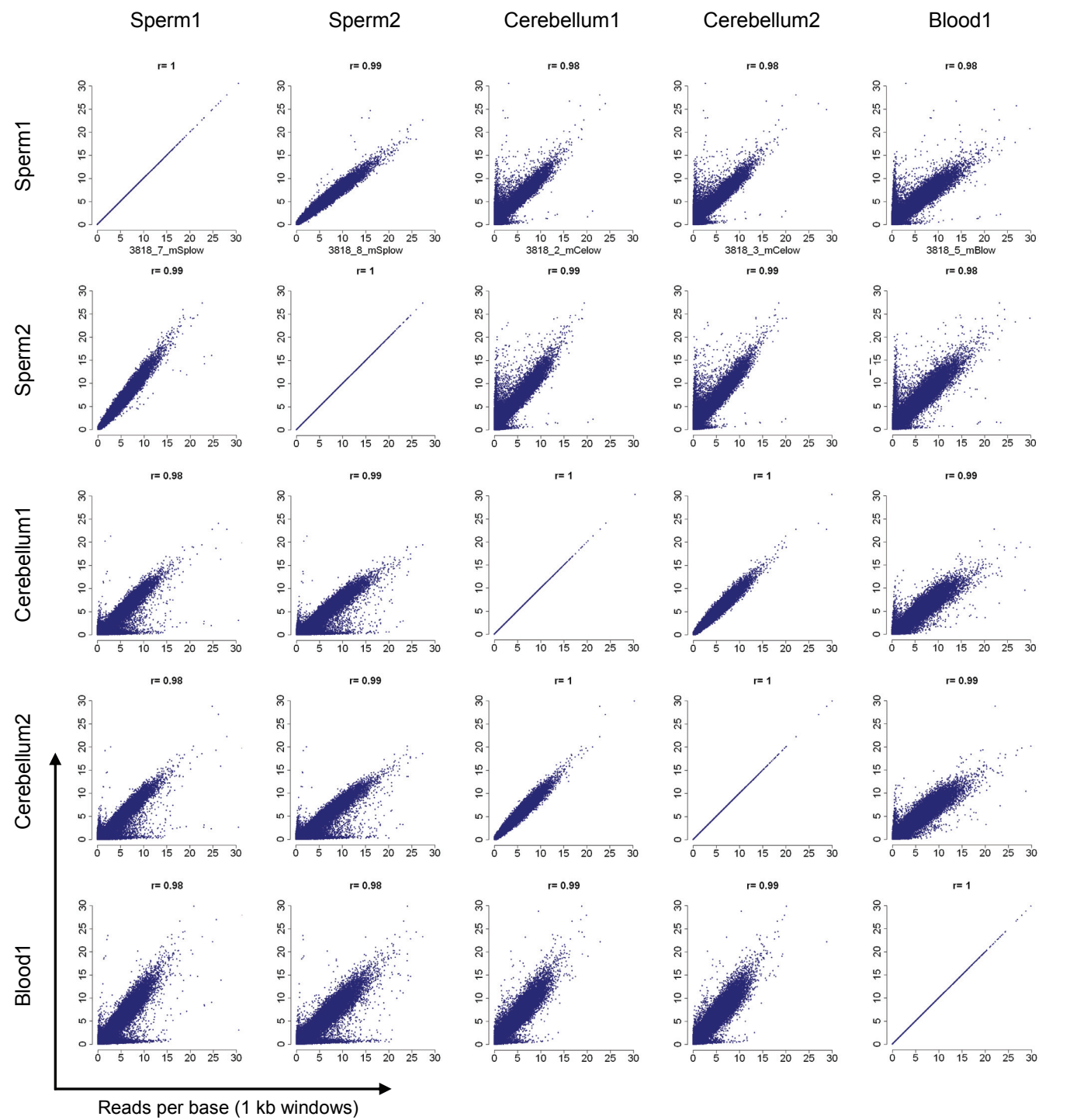

Supplement: Figure S2 — Pairwise analysis of mouse CAP-seq data. Scatter plots of CAP-seq data representing the mean sequence read depth for every contiguous 1 kb window in the mouse genome. Each pairwise comparison was assessed by calculating a Pearson correlation coefficient, which is presented above each plot. Tissue and replicate status for pairwise comparisons are noted above and to the left of the plots. (1.26 MB PDF) [file pgen.1001134.s004.pdf]

Supplementary Figure 3

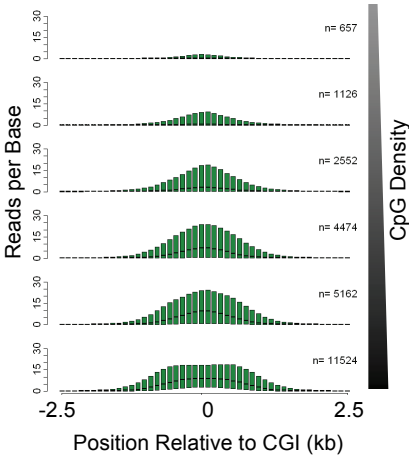

Supplement: Figure S3 — Proportional relationship between CpG density and H3K4me3 at Human CGIs. Box plots of H3K4me3 reads per base (averaged across 500 bp with a 100 bp slide) spanning 5 kb of all human CGIs at different CpG densities (CpGs per 100 bp). CpG density categories applied are ≤5, 5–6, 6–7, 7–8, 8–9 and >9 CpGs per 100 bp, arranged in ascending order from top to bottom. Box plots represent the distribution of the central 50% of the data (filled box) and the median (black bisecting line). The numbers of islands in each category (n) is noted in parenthesis. (0.04 MB PDF) [file pgen.1001134.s005.pdf]

Supplementary Figure 4

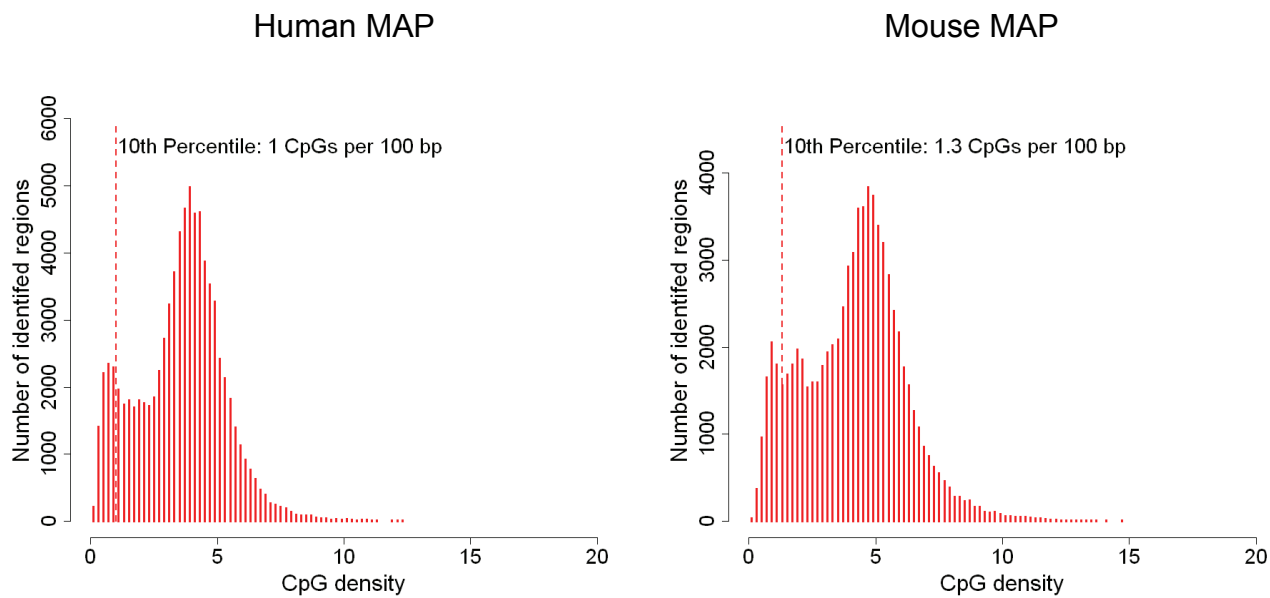

Supplement: Figure S4 — Characterisation of MAP enrichment. Histograms representing the CpG density of MAP-enriched genomic loci in human (hMAP) and mouse (mMAP). The vertical dashed red line represents the lower tenth percentile of the data indicating that the majority of characterised MAP enriched DNA fragments have a CpG density of at least 1 and 1.3 CpGs per 100 bp in human and mouse respectively. (0.05 MB PDF) [file pgen.1001134.s006.pdf]

Supplementary Figure 5

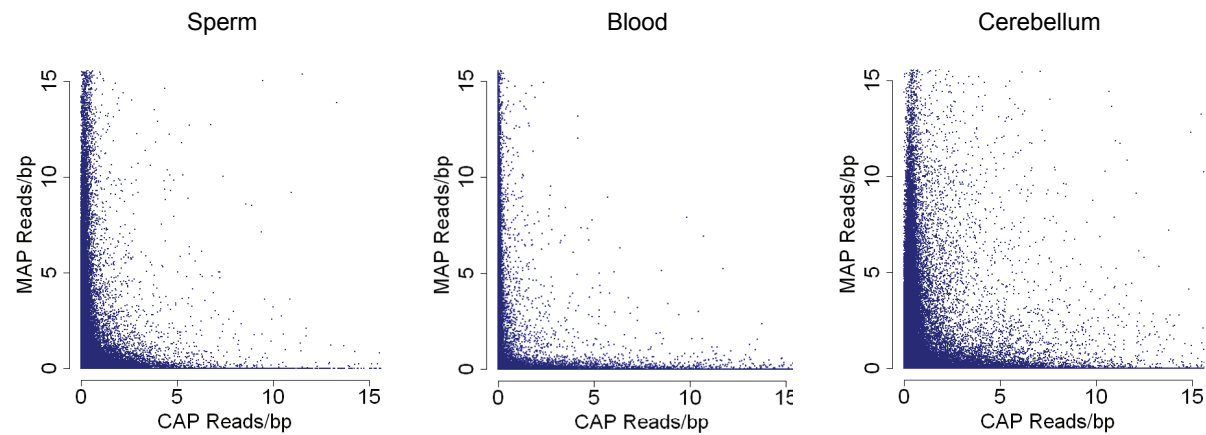

Supplement: Figure S5 — Global scatter plots reveal a reciprocal relationship between CAP- and MAP-seq data for human sperm, blood, and cerebellum. Scatter plots display pairwise comparisons of CAP- and MAP-seq data for every contiguous 1 kb window in the human genome using normalised data for human sperm, blood and cerebellum. Plots are represented as for Figure S2. (0.12 MB PDF) [file pgen.1001134.s007.pdf]

Supplementary Figure 6

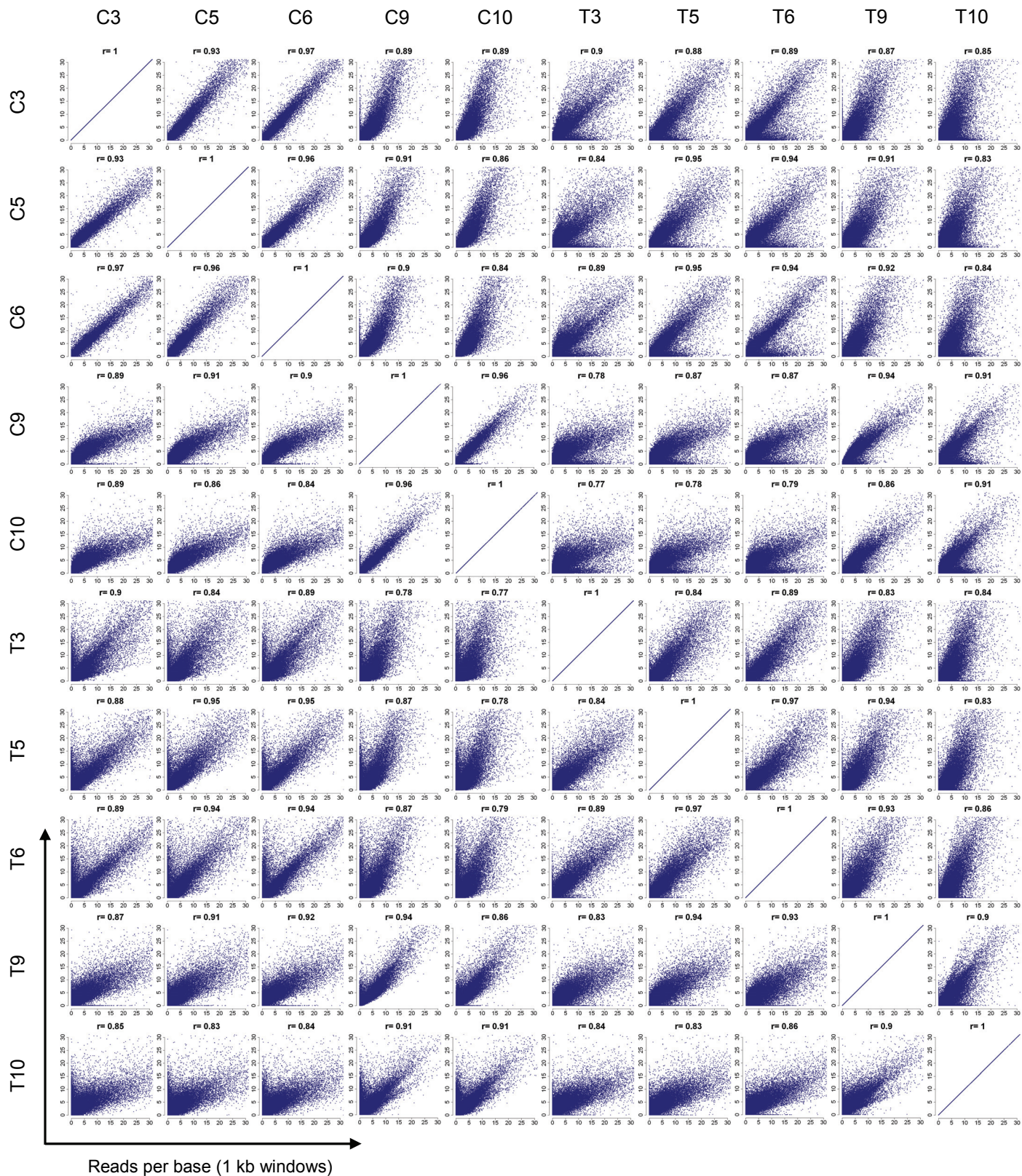

Supplement: Figure S6 — Pairwise comparisons of MAP-seq data reveal consistent tumour-specific methylation. Scatter plots displaying pairwise comparisons of MAP-seq data for every colon (C) and colorectal tumour (T) sample screened by MAP-seq. Data represents the mean sequence depth for every 1 kb window in the human genome. Data is presented as for Figure S2. (4.94 MB PDF) [file pgen.1001134.s008.pdf]

Supplementary Figure 7

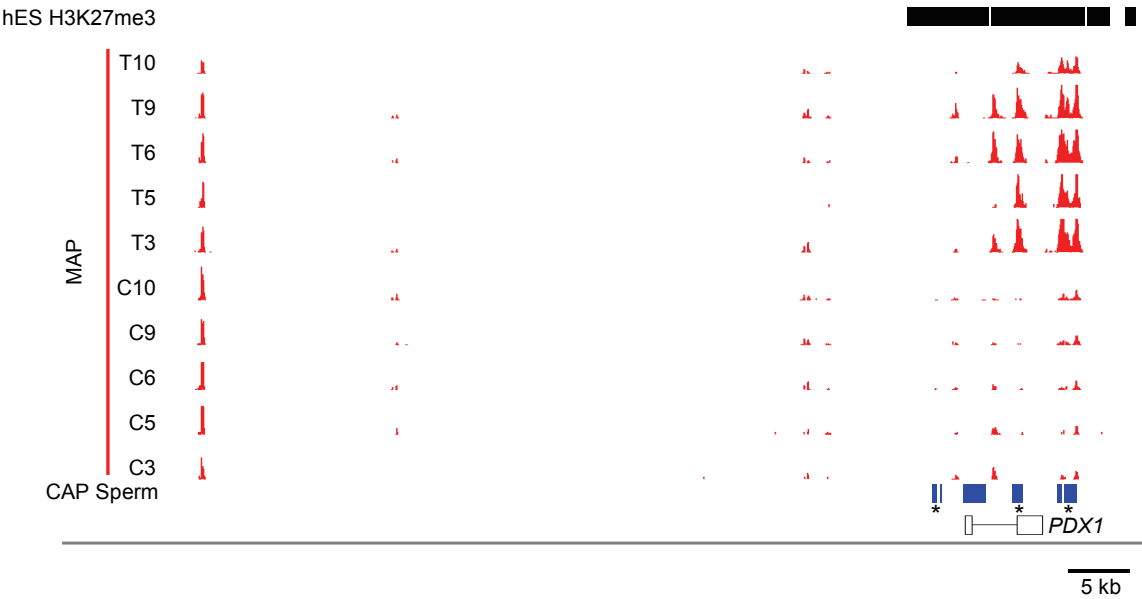

Supplement: Figure S7 — Tumour-specific CGI methylation associated with PDX1. MAP-seq profiles (red) for five colon mucosa (C3, C5, C6, C9 and C10) and five matched colorectal tumour (T3, T5, T6, T9 and T10) biopsy samples for human chr13: 27,325,000–27,402,000. CGIs (blue bars) and sites of hES H3K27 trimethylation (hES H3K27me3; black bars; [46] are represented). (0.05 MB PDF) [file pgen.1001134.s009.pdf]
